# Pancreatic fibrosis, acinar atrophy and chronic inflammation in surgical specimens associated with survival in patients with resectable pancreatic ductal adenocarcinoma

**DOI:** 10.1186/s12885-021-09080-0

**Published:** 2022-01-03

**Authors:** Taija Korpela, Ari Ristimäki, Marianne Udd, Tiina Vuorela, Harri Mustonen, Caj Haglund, Leena Kylänpää, Hanna Seppänen

**Affiliations:** 1 Gastroenterological Surgery, Abdominal Center, Helsinki University Hospital and University of Helsinki, Haartmaninkatu 4, 00029, PL 340 Helsinki, HUS Finland; 2 Department of Pathology, HUSLAB, HUS Diagnostic Center, Helsinki University Hospital and Applied Tumor Genomics Research Program, Research Programs Unit, Faculty of Medicine, University of Helsinki, Helsinki, Finland; 3 Translational Cancer Medicine Research Program, Faculty of Medicine, University of Helsinki and Helsinki University Hospital, Helsinki, Finland

**Keywords:** Pancreatic cancer, Stroma, Tumor–stroma interactions, Chronic pancreatitis

## Abstract

**Background:**

Pancreatic ductal adenocarcinoma **(**PDAC), one of the most lethal malignancies, is increasing in incidence. However, the stromal reaction pathophysiology and its role in PDAC development remain unknown. We, therefore, investigated the potential role of histological chronic pancreatitis findings and chronic inflammation on surgical PDAC specimens and disease-specific survival (DSS).

**Methods:**

Between 2000 and 2016, we retrospectively enrolled 236 PDAC patients treated with curative-intent pancreatic surgery at Helsinki University Hospital. All pancreatic transection margin slides were re-reviewed and ﻿histological findings were evaluated applying international guidelines.

**Results:**

DSS among patients with no fibrosis, acinar atrophy or chronic inflammation identified on pathology slides was significantly better than DSS among patients with fibrosis, acinar atrophy and chronic inflammation [median survival: 41.8 months, 95% confidence interval (CI) 26.0–57.6 vs. 20.6 months, 95% CI 10.3–30.9; log-rank test *p* = 0.001]. Multivariate analysis revealed that Ca 19–9 > 37 kU/l [hazard ratio (HR) 1.48, 95% CI 1.02–2.16], lymph node metastases N1–2 (HR 1.71, 95% CI 1.16–2.52), tumor size > 30 mm (HR 1.47, 95% CI 1.04–2.08), the combined effect of fibrosis and acinar atrophy (HR 1.91, 95% CI 1.27–2.88) and the combined effect of fibrosis, acinar atrophy and chronic inflammation (HR 1.63, 95% CI 1.03–2.58) independently served as unfavorable prognostic factors for DSS. However, we observed no significant associations between tumor size (> 30 mm) and the degree of perilobular fibrosis (*p* = 0.655), intralobular fibrosis (*p* = 0.587), acinar atrophy (*p* = 0.584) or chronic inflammation (*p* = 0.453).

**Conclusions:**

Our results indicate that the pancreatic stroma is associated with PDAC patients’ DSS. Additionally, the more severe the fibrosis, acinar atrophy and chronic inflammation, the worse the impact on DSS, thereby warranting further studies investigating stroma-targeted therapies.

**Supplementary Information:**

The online version contains supplementary material available at 10.1186/s12885-021-09080-0.

## Introduction

Pancreatic ductal adenocarcinoma (PDAC) is one of the leading causes of cancer-related mortality in the Western world with a 5-year relative survival rate of less than 8% [1]. Radical intent pancreatic surgery combined with oncological therapy is typically the only cure for pancreatic cancer (PC) [2, 3]. Yet, only about 10–20% of PDAC patients are suitable for the procedure [4]. In addition, current adjuvant therapies provide only a modest improvement in the overall survival (OS) due to serious chemoresistance [5]. Diabetes mellitus (DM), chronic pancreatitis (CP), cigarette smoking and obesity are known risk factors for PDAC [6–8]. However, the development of PDAC remains poorly understood.

The association between chronic inflammation and cancer development was first recognized a century ago, with evidence increasing in recent years. The short- and long-term risk of PC in CP vary from 2.7 to 13.3 [7, 9–11]. PC risk in CP may result from persistent chronic inflammation in the pancreas [12]. A potential link between chronic inflammation and malignancy was also identified in other gastroenterological conditions, such as inflammatory bowel disease, which is associated with colon cancer [13]. In addition, PC can cause obstructive CP in its immediate vicinity via tumor-related duct obstruction [14]. Mouse models have also confirmed the association between chronic inflammation and PC [15]. Moreover, earlier reports demonstrated an association between preoperatively increased levels of C-reactive protein (CRP) and a worse prognosis [16–19].

In this study, we assessed the potential role of histopathological changes of CP and chronic inflammation in surgical PDAC specimens and their impact on patient survival. Hence, this study aimed to investigate the tumor-related impact on the pancreas outside the tumor bed. In addition, we examined the combination of preoperative CRP, tumor markers and known PC risk factors in relation to the degree of fibrosis, acinar atrophy and chronic inflammation in surgical PDAC specimens.

## Material and methods

### Characteristics of the study population

This retrospective study included 459 patients with PDAC undergoing curative-intent pancreatic surgery at Helsinki University Hospital from 2000 through 2016. Patients undergoing total pancreatectomy (*n* = 12) or a nonanatomical resection (n = 1), ﻿perioperatively deceased (30-d mortality rate, *n* = 2) and lost to follow-up (*n* = 6) were excluded from further analysis. In addition, patients with distal pancreatectomy (*n* = 46) were also excluded since the transection line was on the downstream from PC. We also excluded patients receiving neoadjuvant therapy (*n* = 100) given the potential treatment response in tumor and pancreatic tissues. Furthermore, patients who lacked resection margin tumor specimens (*n* = 42) or whose resection margin specimens were unrepresentative (*n* = 2) were excluded. In total, 236 patients remained for analysis.

Data were collected from medical records and on case-report forms linked to an Access® database. Clinical data included the following parameters: basic demographic characteristics, symptoms of PDAC (jaundice, abdominal or back pain, weight loss and steatorrhea), smoking habits, alcohol intake, inherited genetic syndromes linked to PC, tumor location, treatment modalities (operative details and data on adjuvant chemotherapy), preoperative blood tests [CRP, high-sensitivity CRP (Hs-CRP), serum albumin, Ca 19–9, CEA and total bilirubin], histopathological findings, length of hospital stay, morbidity and follow-up information. The Finnish Population Registry and Statistics Finland provided information on time and cause of death.

### Diagnostic criteria and definitions

The histopathological criteria of CP consisted of fibrosis, loss of acinar tissue (atrophy) and a ductal change according to international guidelines [20]. We also observed any distortion of the ducts and the presence of chronic inflammatory cells. Histological evaluation relied on histological slides from the resection margins. We did not, however, evaluate a diffuse pattern of fibrosis or atrophy since the histological specimens evaluated originated only from the pancreatic transection margin. We intended to investigate the fibrosis and atrophy that was present in the pancreas outside of the tumor bed. Routine slides were primarily stained using hematoxylin and eosin (H&E), some with Herovici.

Pancreatic fibrosis and atrophy were graded according to the scoring system of Klöppel and Maillet [21]. Perilobular fibrosis was defined as ﻿the presence of connective tissue in﻿ the interlobular spaces [21]. Intralobular fibrosis was defined as ﻿the presence of connective tissue extending from the perilobular fibrosis to the acinar lobules with ﻿fibrous replacement of the acinar cells [21]. ﻿Fibrosis was graded as an extension of the fibrosis into the acinar lobules with partial (mild: 10–40%; moderate: 40–80%) or (almost) complete (severe: 80–100%) fibrous replacement of the acinar cells. Acinar atrophy was defined as the destruction of the acinar cells and fibrosis replacement. Acinar atrophy from the tumor specimen slides was similarly graded as follows: partial (mild: 10–40%; moderate: 40–80%) or (almost) complete (severe: 80–100%) fibrous replacement of the acinar cells. Chronic inflammation was graded as mild, moderate and severe according to the number of mononuclear inflammatory cells (see Additional Figs. 1 and 2). Mild and moderate chronic inflammation were characterized by patchy inflammation. Moreover, moderate chronic inflammation exhibited higher numbers of mononuclear inflammatory cells than mild chronic inflammation. A diffuse pattern of inflammation was evident in severe chronic inflammation. Duct changes included ﻿the distortion of ducts, periductal fibrosis, the presence of protein plugs, calculi, epithelial destruction, periductal inflammation and pancreatic intraepithelial neoplasia (PanIN) [21].

### Methods

At surgery, frozen sections were taken to secure a tumor-free margin. However, pancreatic transection margin slides were also taken by the pathologist for the histopathological reporting of carcinomas of the pancreas. The pathology slides were retrieved from the archives and re-reviewed independently by an experienced pathologist (AR) specialized in pancreatology and by the first author (TK) for histological evidence of CP and chronic inflammation. In the event of differing grades, consensus was reached through re-evaluation. The margin clearance was defined as R0 when the distance from the tumor cells to the closest resection margin was > 1 mm and R1 when the distance was ≤1 mm. Tumors were staged according to the Union for International Cancer Control (UICC), 8th edition. A detailed description of the Hs-CRP measurement appears elsewhere [17]. Heavy drinking was defined as three or more drinks daily (1 drink = 125-ml wine, 330-ml beer or 40-ml spirit) almost every day for at least 6 months. The Helsinki University Hospital research board approved the study design (HUS/269/2017), and this study adhered to the Declaration of Helsinki and the International Conference on the Harmonization of Good Clinical Practice.

### Statistical analysis

All calculations and analyses were conducted using IBM’s SPSS version 27 (IBM, SPSS Inc., Chicago, IL, USA). Categorical variables are reported as median (range) or frequency (percent) and compared using the chi-square test or the Fisher’s exact test. Continuous data are reported as means with standard deviations (SDs, for normally distributed data) or as medians and with the interquartile range (IQR). Deviations from the normal distribution were analyzed using the Shapiro–Wilk’s test. The Jonckheere Terpstra test was used to compare differences in continuous variables between ordinal categories. Survival estimates are based on the Kaplan–Meier analysis and log-rank tests. Multivariate analysis was performed using a Cox proportional hazards model. Variables were included in the multivariate analysis based on theoretical importance in order to avoid overfitting the model. Therefore, not all statistically significant variables in the univariate analysis were included in the multivariate analysis. Furthermore, we considered interactions, but found no significant interactions following the Bonferroni correction for multiple comparisons. The assumption of a constant hazard ratio over time was analyzed using the Schoenfeld residuals. We considered *p* < 0.05 as statistically significant.

## Results

### Patient characteristics

Among the 236 patients analyzed (Table 1), 2 (0.8%) had a history of CP and 10 (4.2%) had a history of acute pancreatitis (AP). The median tumor size was 30.0 mm (range, 1.9–75.0 mm). *H. pylori* infection was observed in 8 patients (3.4%), while none had genetic syndromes placing them at an increased risk for PDAC. Gemcitabine was the most-utilized adjuvant chemotherapeutic regimen (Table 1). Altogether, 24 patients (10.2%) received chemotherapy for recurrent PC. Among the 236 patients undergoing a PDAC surgery, 9 patients (3.8%) had a T4 tumor (Table 1). Preoperative imaging assessment failed to detect an unresectable or potentially resectable PC in these patients. The 90-day mortality rate was 1.69% (95% CI 0.05–3.34; *n* = 4). The median hospital stay was 11.0 days (range, 4.0–148.0), and the median follow-up period was 26.8 months (range, 1.4–213.5).

**Table 1:** Baseline characteristics, preoperative clinical and laboratory data and histopathological characteristics of patients with pancreatic ductal adenocarcinoma (PDAC) (*n* = 236)

	*n* = 236
Age at the time of surgery (in years)	67.5 (39.2–85.9)
Sex, male / female	123 (52.1%) / 113 (47.9%)
*ASA class*	
I–II	110 (46.6%)
III–IV	126 (53.4%)
BMI (kg/m^2^) (*n* = 210)	25.3 (15.8–40.1)
*Tobacco smoking (n = 227)*	
Never smoker	115 (48.7%)
Current smoker	46 (19.5%)
Former smoker	58 (24.6%)
*Alcohol consumption (n = 231)*	
Ongoing alcohol misuse	20 (8.5%)
Heavy alcohol intake (ever)	28 (11.9%)
*History of diabetes mellitus*	
No	174 (73.7%)
Yes	62 (26.3%)
Duration of diabetes, in months (*n* = 34)	12.0 (1.0–336.0)
*Blood type*	
A	117 (49.6%)
B	40 (16.9%)
AB	27 (11.4%)
O	52 (22.0%)
*Symptoms*	
Jaundice (n = 235)	184 (78.0%)
Abdominal or back pain (*n* = 235)	105 (44.5%)
Weight loss (n = 234)	96 (40.7%)
Steatorrhea (*n* = 234)	26 (11.0%)
*Preoperative imaging*	
CT	232 (98.3%)
MRCP	67 (28.4%)
Upper abdominal MRI	61 (25.8%)
US	199 (84.3%)
EUS	29 (12.3%)
EUS-FNA	18 (7.6%)
FDG-PET-CT	5 (2.1)
*Preoperative blood test*	
CRP (mg/l) (*n* = 149)	5.0 (1.0–124.0)
High-sensitivity CRP (mg/l) (*n* = 186)	3.4 (0.06–135.5)
Total bilirubin (μmol/l)	17.0 (4.0–511.0)
Ca 19–9 (kU/l) (n = 231)	139.0 (1.0–35,770.0)
CEA (μg/l) (*n* = 229)	2.7 (1.0–68.9)
Albumin (g/l) (n = 234)	36.5 (22.6–46.5)
*Location of pancreatic cancer*	
Head of the pancreas	232 (99.2%)
Body of the pancreas	2 (0.8%)
*T status (n = 235)*	
T1	25 (10.6%)
T2	164 (69.5%)
T3	37 (15.7%)
T4	9 (3.8%)
*LN metastasis (n = 234)*	
N0	62 (26.3%)
N1	171 (72.5%)
N2	1 (0.4%)
*Tumor size (mm) (n = 232)*	
≤30 mm	123 (52.1%)
> 30 mm	109 (46.2%)
Perivascular invasion (*n* = 201)	77 (32.6%)
Perineural invasion (*n* = 216)	174 (73.7%)
*R status (n = 231)*	
R0	178 (75.4%)
R1	55 (22.5%)
Adjuvant chemotherapy	147 (62.3%)

Figures consist of the number of patients (%) or median (range)

Abbreviations: *ASA* American Society of Anesthesiologists; *BMI* body mass index; *CT* computed tomography; *DM* diabetes mellitus; *EUS* endoscopic ultrasound; *EUS-FNA* endoscopic ultrasound-guided fine needle aspiration biopsy; *FDG-PET-CT* fluorodeoxyglucose (FDG) positron emission tomography (PET); *MRCP* magnetic resonance cholangiopancreatography; *MRI* magnetic resonance imaging; *US* ultrasound

The fibrosis grades and inflammation activity scores appear in Table 2. Across all 236 patients, PanIN-1A was detected in 64 patients (27.1%), PanIN-2 in 5 (2.1%), PanIN-1B in 3 (1.3%), serous cystadenoma in 1 (0.4%) and high-grade dysplasia in 1 patient (0.4%).

**Table 2:** Grade of fibrosis and chronic inflammation in patients with pancreatic ductal adenocarcinoma (PDAC) (*n* = 236)

	Mild	Moderate	Severe	No fibrosis, atrophy or chronic inflammation
*Pattern of fibrosis*				
Perilobular fibrosis	35 (14.8%)	55 (23.3%)	119 (50.4%)	26 (11.0%)
Intralobular fibrosis	44 (18.6%)	54 (22.9%)	94 (39.8%)	43 (18.2%)
Acinar atrophy	29 (12.3%)	47 (19.9%)	103 (43.6%)	57 (24.2%)
Chronic inflammation	92 (39.0%)	55 (23.3%)	5 (2.1%)	84 (35.6%)

Figures consist of number of patients (%)

### Survival analysis of prognostic factors for DSS

**Fig. 1:**
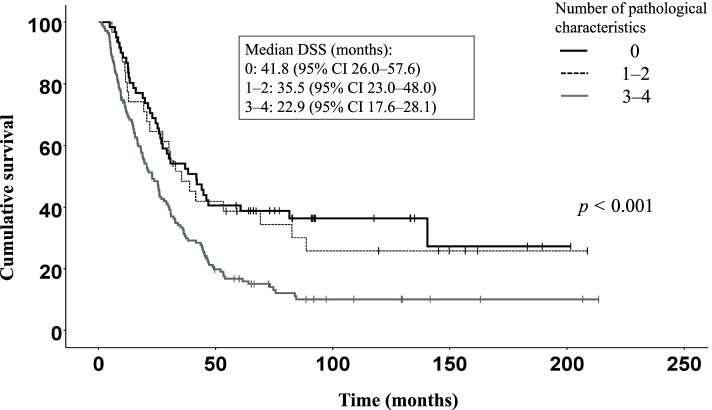
Combined effect of perilobular and intralobular fibrosis, acinar atrophy and chronic inflammation (pathological characteristics). Kaplan–Meier overall survival curve stratified by the number of pathological characteristics a patient exhibited in the histological assessment. The overall log-rank (*p* < 0.001), between 0 and 3–4 pathological characteristics (*p* < 0.001) and between 1 and 2 and 3–4 pathological charateristics (*p* = 0.014). We used the Bonferroni correction for multiple comparisons with the decision level set to *p* < 0.025

**Fig. 2:**
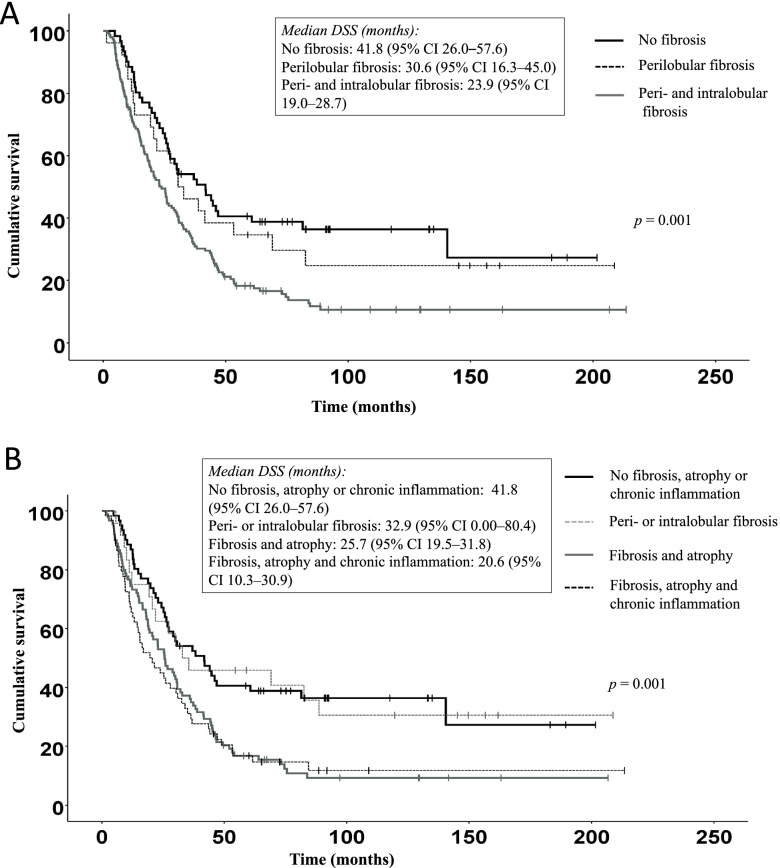
Kaplan–Meier curves showing the combined effects of (**A**) perilobular and intralobular fibrosis and (**B**) perilobular and intralobular fibrosis, acinar atrophy and chronic inflammation. **A** The overall log-rank (*p* = 0.001), between no fibrosis and peri- and intralobular fibrosis (*p* < 0.001) and between perilobular fibrosis and perilobular and intralobular fibrosis (*p* = 0.072). **B** The overall log-rank (*p* = 0.001), a comparison between no fibrosis, acinar athropy or chronic inflammation versus peri- or intralobular fibrosis (*p* = 0.914), versus fibrosis and atrophy (*p* = 0.001) or versus fibrosis, acinar atrophy and chronic inflammation (*p* = 0.001). We used the Bonferroni correction for multiple comparisons, with the decision level set to at *p* < 0.025 in A and *p* < 0.017 in B

Using Kaplan–Meier curves, perilobular and intralobular fibrosis, acinar atrophy and chronic inflammation significantly associated with DSS (Figs. 1 and 2). Subgroup survival outcomes according to perilobular and intralobular fibrosis, acinar atrophy and chronic inflammation appear in Figs. 1 and 2.

Figure 1 shows DSS stratified by the number of pathological variables (perilobular and intralobular fibrosis, acinar atrophy and chronic inflammation) a patient exhibited in the histological analysis. Figure 2A and B show DSS stratified by the combined effects of perilobular and intralobular fibrosis, acinar atrophy and chronic inflammation.

DSS among patients with one or two pathological variables was significantly better than DSS among patients with three or four pathological variables [log-rank test, *p* = 0.014; median survival: 35.5 months (95% CI 23.0–48.0) vs. 22.9 months (95% CI 17.6–28.1); Fig. 1). Figure 1 shows the results from the overall log-rank test (*p* < 0.001), as well as a comparison between no pathological variables and three or four pathological variables (*p* < 0.001) and a comparison of one or two pathological variables versus three or four pathological variables (*p* = 0.014).

In addition, DSS among patients with no fibrosis was significantly better than DSS among patients with perilobular and intralobular fibrosis [log-rank test, *p* = 0.004; median survival: 41.8 months (95% CI 26.0–57.6) vs. 23.9 months (95% CI 19.0–28.7); Fig. 2A). Figure 2A shows the results from the overall log-rank test (*p* = 0.001), as well as a comparison between no fibrosis versus peri- and intralobular fibrosis (*p* < 0.001), and a comparison between perilobular fibrosis versus perilobular and intralobular fibrosis (*p* = 0.072).

We also found that DSS among patients with no fibrosis, atrophy or chronic inflammation was significantly better than DSS among patients with fibrosis, atrophy and chronic inflammation [log-rank *p* = 0.001; median survival: 41.8 months (95% CI 26.0–57.6) vs. 20.6 months (95% CI 10.3–30.9); Fig. 2B). Figure 2B shows the results from the overall log-rank test (*p* = 0.001), as well as a comparison between no fibrosis, acinar athropy or chronic inflammation versus peri- or intralobular fibrosis (*p* = 0.914) and a comparison between fibrosis and atrophy (*p* = 0.001) or fibrosis, acinar atrophy and chronic inflammation (*p* = 0.001).

Table 3 identifies those variables that correlated with DSS. In our univariate analysis, we found that patients with a preoperative albumin > 36 g/l exhibited a better survival (*p* = 0.011). Ca 19–9 > 37 kU/l (*p* = 0.002), CEA > 5.0 μg/l (*p* = 0.027) and bilirubin > 20 μmol/l (*p* = 0.030) associated with significantly higher hazard ratios (HRs) and emerged as prognostic factors for a worse DSS. Furthermore, T stage T3–4 (*p* = 0.029), lymph node metastases N1–2 (*p* = 0.001), tumor grade 3 (*p* = 0.017), a tumor > 30 mm (*p* < 0.000) all associated with a worse prognosis. In addition, severe perilobular fibrosis (*p* = 0.013), moderate and severe intralobular fibrosis (*p* = 0.033 and *p* = 0.002), moderate and severe atrophy (*p* = 0.004 and *p* = 0.001) and mild and moderate chronic inflammation (*p* = 0.010 and *p* = 0.002) all emerged as unfavorable prognostic factors for DSS.

**Table 3:** Univariate and multivariate analysis of disease-specific survival (DSS) among PDAC patients (n = 236)

Variables	Univariate analysis		Multivariate analysis	
	HR (95% CI)	*p*	HR (95% CI)	*p*
Age, > 65 years	1.14 (0.85–1.53)	0.389	0.98 (0.71–1.35)	0.883
Gender, female / male	1.12 (0.84–1.50)	0.425	0.91 (0.66–1.26)	0.573
ASA score				
I–II	1 (ref)	–	1 (ref)	–
III–IV	1.04 (0.78–1.38)	0.815	0.96 (0.69–1.35)	0.830
Blood group A, B or AB	1.22 (0.86–1.73)	0.275		
Preoperative blood test				
Ca 19–9 ≥ 37 kU/l	1.75 (1.24–2.48)	0.002	1.48 (1.02–2.16)	0.040
CEA > 5.0 μg/l	1.53 (1.05–2.22)	0.027	1.24 (0.84–1.83)	0.285
Albumin > 36 g/l	0.69 (0.51–0.92)	0.011	0.76 (0.56–1.05)	0.091
Bilirubin > 20 μmol/l	1.38 (1.03–1.84)	0.030		
CRP (mg/l)				
CRP > 5 mg/l	1.75 (1.20–2.55)	0.004		
log CRP	1.90 (1.20–3.01)	0.006		
Hs-CRP > 3 mg/l	1.56 (1.12–2.18)	0.009		
log Hs-CRP	1.64 (1.22–2.20)	0.001		
T stage				
T1–2	1 (ref)	–	1 (ref)	–
T3–4	1.48 (1.04–2.10)	0.029	0.98 (0.64–1.50)	0.922
N stage				
N0	1 (ref)	–	1 (ref)	–
N1–2	1.85 (1.30–2.63)	0.001	1.71 (1.16–2.52)	0.007
Tumor grading				
1	1 (ref)	–	1 (ref)	–
2	1.08 (0.69–1.70)	0.741		
3	1.99 (1.13–3.49)	0.017		
Tumor size (mm)				
≤ 30 mm	1 (ref)	–	1 (ref)	–
> 30 mm	1.72 (1.28–2.30)	0.000	1.47 (1.04–2.08)	0.031
Perivascular invasion	1.21 (0.49–3.00)	0.680		
Perineural invasion	1.96 (0.88–4.35)	0.100		
Adjuvant treatment	1.01 (0.98–1.05)	0.407		
Perilobular fibrosis				
No fibrosis	1 (ref)	–		
Mild fibrosis	0.98 (0.52–1.84)	0.939		
Moderate fibrosis	1.44 (0.82–2.52)	0.201		
Severe fibrosis	1.92 (1.15–3.21)	0.013		
Intralobular fibrosis				
No fibrosis	1 (ref)	–		
Mild fibrosis	1.06 (0.63–1.78)	0.824		
Moderate fibrosis	1.67 (1.04–2.69)	0.033		
Severe fibrosis	1.95 (1.27–3.01)	0.002		
Acinar atrophy				
No atrophy	1 (ref)	–		
Mild atrophy	1.03 (0.59–1.79)	0.924		
Moderate atrophy	1.92 (1.23–3.00)	0.004		
Severe atrophy	1.92 (1.30–2.82)	0.001		
Chronic inflammation				
No chronic inflammation	1 (ref)	–		
Mild chronic inflammation	1.57 (1.11–2.21)	0.010		
Moderate chronic inflammation	1.85 (1.26–2.74)	0.002		
Severe chronic inflammation	1.90 (0.69–5.25)	0.214		
Combined effects of fibrosis, atrophy and chronic inflammation (Fig. 2b)				
No fibrosis, atrophy and chronic inflammation	1 (ref)	–	1 (ref)	–
Peri- or intralobular fibrosis	1.03 (0.57–1.84)	0.929	1.04 (0.56–1.93)	0.893
Fibrosis and atrophy	1.89 (1.28–2.77)	0.001	1.91 (1.27–2.88)	0.002
Fibrosis, atrophy and inflammation	2.03 (1.33–3.09)	0.001	1.63 (1.03–2.58)	0.038

Abbreviation: *ASA* American Society of Anesthesiologists

Next, we performed a multivariate analysis to identify prognostic factors associated with DSS (Table 3). Ca 19–9 > 37 kU/l (*p* = 0.040), lymph node metastases N1–2 (*p* = 0.007) and a tumor > 30 mm (*p* = 0.031) all emerged as unfavorable prognostic factors for DSS. In addition, the combined effect of fibrosis and acinar atrophy (*p* = 0.002) and fibrosis, acinar atrophy and chronic inflammation (*p* = 0.038) emerged as unfavorable prognostic factors for DSS (Fig. 2B).

We also analyzed the relationship between the tumor size and the degree of fibrosis, acinar atrophy and chronic inflammation. Interestingly, tumor size (> 30 mm) did not associate with perilobular fibrosis (no and mild perilobular fibrosis vs. moderate and severe perilobular fibrosis) [*n* = 27 (44.3%) vs. *n* = 82 (48.2%); *p* = 0.655], intralobular fibrosis (no and mild intralobular fibrosis vs. moderate and severe intralobular fibrosis) [*n* = 38 (44.7%) vs. *n* = 71 (48.6%); *p* = 0.587], acinar atrophy (no and mild atrophy vs. moderate and severe atrophy) [*n* = 37 (44.0%) vs. *n* = 72 (48.6%); *p* = 0.584] or chronic inflammation (no and mild chronic inflammation vs. moderate and severe chronic inflammation) [*n* = 78 (45.3%) vs. *n* = 31 (51.7%); *p* = 0.453]. In addition, we investigated the relationship between the tumor grade (1–3) and the degree of fibrosis, acinar atrophy and chronic inflammation. We found no statistically significant association between the tumor grade (1–3) and the degree of fibrosis or in any of the subgroups: perilobular fibrosis (no and mild perilobular fibrosis vs. moderate and severe perilobular fibrosis; *p* = 0.904), intralobular fibrosis (no and mild intralobular fibrosis vs. moderate and severe intralobular fibrosis; *p* = 0.477), acinar atrophy (no and mild atrophy vs. moderate and severe atrophy; *p* = 0.516) or chronic inflammation (no and mild chronic inflammation vs. moderate and severe chronic inflammation; *p* = 0.225) (see Additional Table 1).

### CRP and high-sensitivity CRP

In our univariate analysis, we found that patients with a CRP > 5 mg/l (*p* = 0.004), log CRP (*p* = 0.006), Hs-CRP > 3 mg/l (*p* = 0.009) and log Hs-CRP (*p* = 0.001) all exhibited a worse DSS (Table 3). However, given the number of missing values, CRP (*n* = 87) and Hs-CRP (*n* = 50) were unsuitable for further multivariate analysis.

We also analyzed the association between preoperative blood tests and perilobular and intralobular fibrosis, acinar atrophy and chronic inflammation (Table 4). We found a statistically significant association between the degree of perilobular fibrosis and CEA (*p* = 0.002) and albumin (*p* = 0.027) (Table 4). In addition, we found a statistically significant association between the degree of intralobular fibrosis and CEA (*p* = 0.002; Table 4). We also identified a statistically significant association between the degree of acinar atrophy and CEA (*p* = 0.002) and albumin (*p* = 0.009; Table 4). Similarly, a statistically significant association emerged between the degree of chronic inflammation and CEA (*p* = 0.004) and albumin (*p* = 0.007; Table 4). However, we found no association between tumor size and perilobular (*p* = 0.701) and intralobular (*p* = 0.556) fibrosis, acinar atrophy (*p* = 0.338) or chronic inflammation (*p* = 0.231; Table 4).

**Table 4:** Results from the Jonckheere Terpstra test evaluating the association between preoperative blood tests and tumor size, and perilobular and intralobular fibrosis, atrophy and chronic inflammation

	No perilobular fibrosis, median (IQR)	Mild perilobular fibrosis, median (IQR)	Moderate perilobular fibrosis, median (IQR)	Severe perilobular fibrosis, median (IQR)	*p*
CRP (mg/l)	5.0 (7.5)	3.0 (7.5)	5.0 (7.5)	5.0 (7.5)	0.480
High-sensitivity CRP (mg/l)	5.8 (8.4)	4.0 (8.4)	3.7 (8.4)	2.9 (8.4)	0.246
Ca 19–9 (kU/l)	129.0 (556.0)	82.5 (556.0)	176.5 (556.0)	126.0 (556.0)	0.454
CEA (μg/l)	2.2 (2.7)	2.5 (2.7)	2.6 (2.7)	3.0 (2.7)	0.002
Albumin (g/l)	37.4 (5.4)	36.9 (5.4)	37.3 (5.4)	36.2 (5.4)	0.027
Tumor size (mm)	32.0 (15.0)	30.0 (15.0)	35.0 (15.0)	30.0 (15.0)	0.701
	No intralobular fibrosis, median (IQR)	Mild intralobular fibrosis, median (IQR)	Moderate intralobular fibrosis, median (IQR)	Severe intralobular fibrosis, median (IQR)	*p*
CRP (mg/l)	5.5 (7.5)	3.0 (7.5)	4.5 (7.5)	5.0 (7.5)	0.546
High-sensitivity CRP (mg/l)	5.2 (8.4)	4.1 (8.4)	3.2 (8.4)	3.1 (8.4)	0.403
Ca 19–9 (kU/l)	88.0 (556.0)	147.0 (556.0)	184.0 (556.0)	158.5 (556.0)	0.088
CEA (μg/l)	2.0 (2.7)	2.7 (2.7)	2.8 (2.7)	3.0 (2.7)	0.002
Albumin (g/l)	37.3 (5.4)	36.4 (5.4)	37.7 (5.4)	36.2 (5.4)	0.074
Tumor size (mm)	30.0 (15.0)	30.0 (15.0)	35.0 (15.0)	30.0 (15.0)	0.556
	No acinar atrophy, median (IQR)	Mild acinar atrophy, median (IQR)	Moderate acinar atrophy, median (IQR)	Severe acinar atrophy, median (IQR)	*p*
CRP (mg/l)	5.0 (7.5)	4.0 (7.5)	4.0 (7.5)	5.0 (7.5)	0.431
High-sensitivity CRP (mg/l)	4.9 (8.4)	4.1 (8.4)	3.4 (8.4)	2.6 (8.4)	0.268
Ca 19–9 (kU/l)	124.0 (556.0)	74.0 (556.0)	168.0 (556.0)	216.5 (556.0)	0.058
CEA (μg/l)	2.5 (2.7)	2.5 (2.7)	2.6 (2.7)	3.1 (2.7)	0.002
Albumin (g/l)	37.2 (5.4)	36.4 (5.4)	37.6 (5.4)	36.1 (5.4)	0.009
Tumor size (mm)	30.0 (15.0)	30.0 (15.0)	35.0 (15.0)	30.0 (15.0)	0.338
	No chronic inflammation, median (IQR)	Mild chronic inflammation, median (IQR)	Moderate chronic inflammation, median (IQR)	Severe chronic inflammation, median (IQR)	*p*
CRP (mg/l)	4.5 (7.5)	4.0 (7.5)	6.0 (7.5)	4.0 (7.5)	0.328
High-sensitivity CRP (mg/l)	3.2 (8.4)	3.1 (8.4)	3.5 (8.4)	2.8 (8.4)	0.964
Ca 19–9 (kU/l)	121.0 (556.0)	173.0 (556.0)	184.0 (556.0)	322.0 (556.0)	0.167
CEA (μg/l)	2.5 (2.7)	2.7 (2.7)	3.4 (2.7)	2.4 (2.7)	0.004
Albumin (g/l)	37.1 (5.4)	37.0 (5.4)	35.9 (5.4)	34.9 (5.4)	0.007
Tumor size (mm)	30.0 (15.0)	30.0 (15.0)	33.0 (15.0)	25.0 (15.0)	0.231

Abbreviation: *IQR* interquartile range

### Mortality

A total of 197 (83.5%) patients died during the median follow-up of 26.8 months. PC was listed as the cause of death in 186 patients (94.4%). Other causes of death included intracranial hemorrhage in 2 patients (1.0%), heart disease in 2 (1.0%), stroke in 2 (1.0%), lung cancer in 1 (0.5%), Alzheimer’s disease in 1 (0.5%), pneumonia in 1 (0.5%), suicide in 1 (0.5%) and undetermined in 1 patient (0.5%). The median overall survival estimate was 26.8 months (95% CI 22.6–31.1) and DSS was 27.4 months (95% CI 23.4–31.3).

## Discussion

In this study, we evaluated the association between histological CP findings and chronic inflammation in surgical PDAC specimens on DSS. Our results suggest that patients with no fibrosis, atrophy or chronic inflammation found during histopathological analysis exhibit a significantly better DSS than patients with fibrosis, atrophy and chronic inflammation (41.8 months vs. 20.6 months; Fig. 2B). Furthermore, the more profound the severity of fibrosis, atrophy and chronic inflammation, the worse the impact on DSS. In addition, Ca 19–9 > 37 kU/l, lymph node metastases N1–2, tumor size > 30 mm and the combined effect of fibrosis, acinar atrophy and chronic inflammation all served as unfavorable prognostic factors for DSS. However, we observed no significant associations between tumor size and the degree of fibrosis, acinar atrophy or chronic inflammation. Moreover, the tumor grade did not associate with the degree of fibrosis.

Previous studies indicated an ability of pancreatic cancer cells to recruit stromal cells to produce a growth-favorable environment by promoting tumor proliferation, invasion, metastasis and chemoresistance [22, 23]. However, the relationship and the molecular mechanisms between stroma and pancreatic cancer cells remains incompletely understood [24]. In this study, we assessed the role and characteristics of pancreatic stroma on survival among patients with resectable PDAC. The subgroup analyses in our study suggest that patients with no fibrosis found during histopathological analysis enjoy a significantly better DSS than patients with perilobular and intralobular fibrosis (41.8 months vs. 23.9 months; Fig. 2A). In current clinical practice, patients with resected pancreatic cancer receive adjuvant treatment [2, 25]. However, among our study population we found no association between adjuvant treatment and DSS (HR 1.01, 95% CI 0.98–1.05). A retrospective cohort study among 66 PDAC patients undergoing pancreaticoduodenectomy treated with adjuvant therapy ﻿utilized a computer-aided method to assess the density and activity of the stroma [26]. In that study, a high stromal density in resected PDAC patients associated with longer disease-free and overall survival. Similarly, a study among two cohorts of 400 patients with sporadic PDAC examined the tumor–stroma ratio using digitalized whole-mount slide images, observing that intratumoral necrosis and R1 independently associated with a low stromal component in the developing cohort (207 patients) [27]. Conversely, in a study by Bolm et al. [28], the stroma density was not associated with tumor progression or OS. In our study, we identified a synergy between perilobular and intralobular fibrosis, acinar atrophy and chronic inflammation indicative of a worse survival (Fig. 1). Among patients with one or two pathological characteristics (perilobular and intralobular fibrosis, acinar atrophy or chronic inflammation) found during histopathological analysis, DSS was significantly better than among patients with three or four pathological characteristics (35.5 months vs. 22.9 months; Fig. 1).

Currently, evidence indicates that ﻿systemic and intrapancreatic inflammation plays a major role in the development and progression ﻿of PC [29]. A prolonged inflammatory response can result from ﻿CP, alcohol consumption, DM, ﻿hereditary pancreatitis, obesity and cigarette smoking [29, 30]. Among our study population, chronic inflammation was apparent in the surgical PDAC specimen from 152 patients (64.4%; Table 2). Previous reports demonstrated that > 90% of patients with PDAC have mutations in the KRAS gene; thus, as shown in mouse models, activation of the oncogenic KRAS necessitates chronic inflammation [15, 31, 32]. In our study, patients with mild (*p* = 0.010) or moderate (*p* = 0.002) chronic inflammation found during histopathological analysis exhibited a worse DSS than patients with no chronic inflammation. This observation may indicate the important role of inflammation in PDAC progression.

In addition, the presence of a systemic inflammatory response syndrome in cancer patients predicts a poor outcome [33, 34]. Moreover, the study by Knoop et al. [35] demonstrated in mouse models that significantly improving OS with gemcitabine was abolished by CP and a systemic inflammatory response syndrome. Similarly, a prospective cohort study among ﻿61,597 healthy subjects with an 18-year follow-up period found evidence of a positive association between serum haptoglobin, CRP and leukocytes and the risk of developing PC [36]. Here, we demonstrated that patients with CRP > 5 mg/l (*p* = 0.004) and Hs-CRP > 3 mg/l (*p* = 0.009) experienced a worse DSS (Table 3). However, no association emerged between the CRP levels and perilobular fibrosis, intralobular fibrosis, acinar atrophy or chronic inflammation (Table 4). Yet, we found a statistically significant association between CEA and the degree of perilobular fibrosis (*p* = 0.002), intralobular fibrosis (*p* = 0.002), acinar atrophy (*p* = 0.002) and chronic inflammation (*p* = 0.004; Table 4). Additionally, we observed a similar association between albumin and perilobular fibrosis (*p =* 0.027), acinar atrophy (*p =* 0.009) and chronic inflammation (*p =* 0.007). However, in a multivariate analysis, a high CEA value did not significantly associate with DSS (*p* = 0.285). This result differs markedly from previous studies that observed a worse prognosis in PDAC patients with high preoperative CEA values [37, 38]. We argue that a high CEA value indicates a worse DSS and associates with the degree of fibrosis, acinar atrophy and chronic inflammation.

Previous studies indicated that PC causes pancreatitis through tumor-related duct obstruction [39–41]. Hence, the tumor-associated ductal obstruction could also induce severe fibrosis and acinar atrophy. In contrast, we found no association between a tumor size > 30 mm and any of the following: moderate and severe perilobular fibrosis (*p* = 0.655), moderate and severe intralobular fibrosis (*p* = 0.587), moderate and severe acinar atrophy (*p* = 0.584) or moderate and severe chronic inflammation (*p* = 0.453). A cohort study among ﻿12,522 Danish patients and 37,552 US patients with PC revealed that ﻿patients with AP diagnosed 90 days before a PC diagnosis exhibited a lower tumor stage, higher resection frequencies and better survival [41]. In our study, however, only 10 patients (4.2%) had a history of AP.

One of the strengths of our study is that all pancreatic transection margin slides were re-reviewed and graded by an experienced pathologist (AR) specialized in pancreatology and by the first author (TK). In addition, the number of PDAC patients in our study was large. That said, our study also carries several limitations. First, we relied on a retrospective cohort study design. Second, histological evaluation relied on histological slides from the resection margins and we did not evaluate the entire surgical specimen. We examined histological changes only for the transection margin slides since the aim was to evaluate the tumor-free area and the pancreatic stroma. Furthermore, there is no certainty that the resection margin fell within a similar distance to the tumor bed in all of the pathology slides. Thus, we evaluated the presence of pancreatic cancer cells in all of the pathology slides. Third, we could not obtain preoperative CRP and Hs-CRP values for all PDAC patients undergoing surgery. However, the information provided from our study strengthens the prognostic value of the stroma in resected PDACs, warranting further study.

## Conclusions

In conclusion, our results indicate that the degree of fibrosis, acinar atrophy and chronic inflammation serve as prognostic factors in resectable PDAC patients. In addition, we observed a combined effect for several pathological characteristics that serve as unfavorable prognostic factors for DSS. Moreover, this study provides evidence of the prognostic value of the stroma on PDAC patient survival. We also established the association between CEA and perilobular and intralobular fibrosis, acinar atrophy and chronic inflammation. Interestingly, we found no association between tumor size (> 30 mm) and the degree of fibrosis, acinar atrophy or chronic inflammation. Further research is required to determine the prognostic value of the stroma in resectable PDAC patients and potential new stroma-targeting therapeutic strategies.

## Supplementary Information

**Additional file 1.**

**Additional file 2.**

**Additional file 3.**

## Data Availability

The data that support the findings of this study are not publicly available due to institutional regulations, However, the data are available from the corresponding author upon reasonable request.

## Abbreviations

ASA: American Society of Anesthesiologists grade
AP: Acute pancreatitis
BMI: Body mass index
CI: Confidence interval
CP: Chronic pancreatitis
CRP: C-reactive protein
CT: Computed tomography
DM: Diabetes mellitus
DSS: Disease-specific survival
EUS: Endoscopic ultrasound
EUS-FNA: Endoscopic ultrasound-guided fine-needle aspiration biopsy
FDG-PET-CT: Fluorodeoxyglucose (FDG) positron emission tomography (PET)
HR: Hazard ratio
Hs-CRP: High-sensitivity CRP
IQR: Interquartile range
MRCP: Magnetic resonance cholangiopancreatography
MRI: Magnetic resonance imaging
OS: Overall survival
PanIN: Pancreatic intraepithelial neoplasia
PC: Pancreatic cancer
PDAC: Pancreatic ductal adenocarcinoma
US: Ultrasound
