# Supplementary material for: Pancreatic fibrosis, acinar atrophy and chronic inflammation in surgical specimens associated with survival in patients with resectable pancreatic ductal adenocarcinoma
Source: BMC Cancer. 2022 Jan 3;22:23. doi: 10.1186/s12885-021-09080-0 (PMC8721973; doi:10.1186/s12885-021-09080-0)
Supplement: Supplementary file 1 — Additional file 1. [file 12885_2021_9080_MOESM1_ESM.pdf]

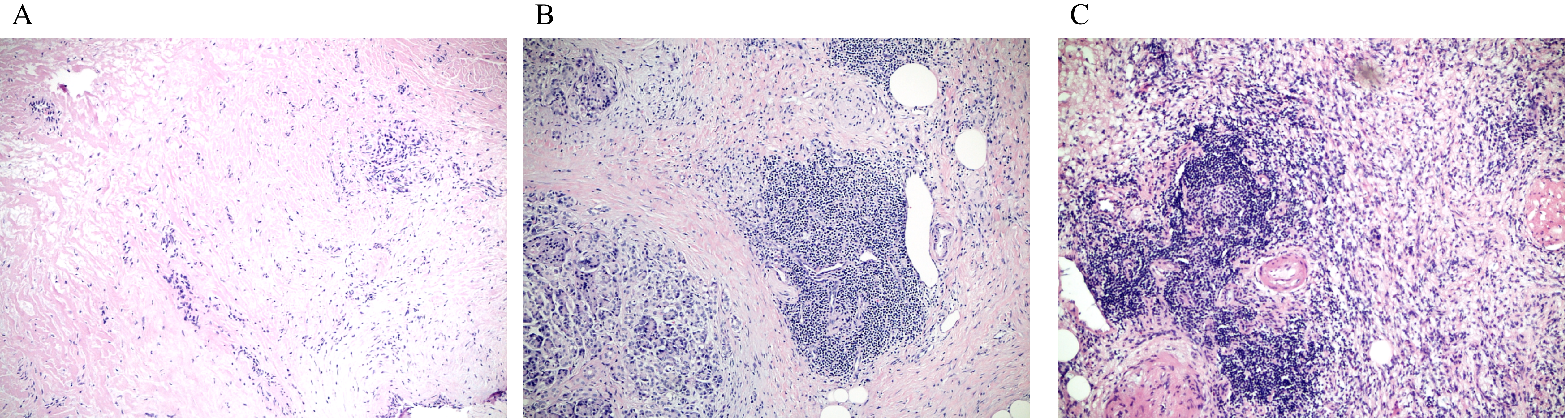

Additional Figure 1. Histological presentation of chronic inflammation. A. Mild chronic inflammation (H&E stain; 100X). B. Moderate chronic inflammation (H&E stain; 100X). C. Severe chronic inflammation (H&E stain; 100X).
