# Supplementary material for: Pancreatic fibrosis, acinar atrophy and chronic inflammation in surgical specimens associated with survival in patients with resectable pancreatic ductal adenocarcinoma
Source: BMC Cancer. 2022 Jan 3;22:23. doi: 10.1186/s12885-021-09080-0 (PMC8721973; doi:10.1186/s12885-021-09080-0)
Supplement: Supplementary file 2 — Additional file 2. [file 12885_2021_9080_MOESM2_ESM.pdf]

A B C

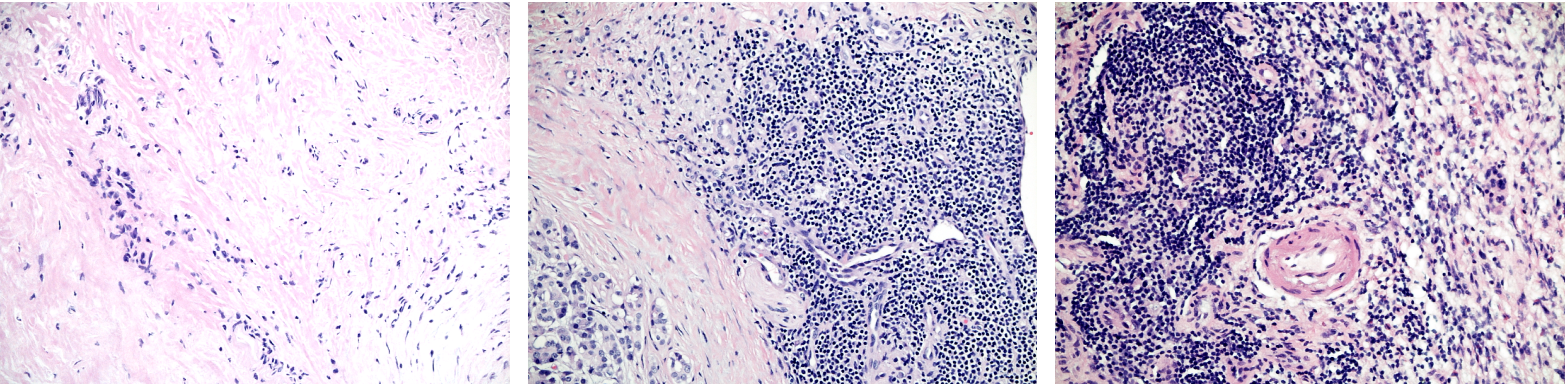

Additional Figure 2. Histological presentation of chronic inflammation. A. Mild chronic inflammation (H&E stain; 200X). B. Moderate chronic inflammation (H&E stain; 200X). C. Severe chronic inflammation (H&E stain; 200X).
