# Supplementary material for: Pancreatic fibrosis, acinar atrophy and chronic inflammation in surgical specimens associated with survival in patients with resectable pancreatic ductal adenocarcinoma
Source: BMC Cancer. 2022 Jan 3;22:23. doi: 10.1186/s12885-021-09080-0 (PMC8721973; doi:10.1186/s12885-021-09080-0)
Supplement: Supplementary file 3 — Additional file 3. [file 12885_2021_9080_MOESM3_ESM.docx]

**SUPPLEMENTARY MATERIAL**

**Additional Table 1.** Crosstabs showing the association between the tumor grade (1–3) and the degree of fibrosis, acinar atrophy and chronic inflammation.

|  | Tumor grade | | |
| --- | --- | --- | --- |
|  | 1 | 2 | 3 |
| *Perilobular fibrosis* (*p* = 0.904) | | | |
| No and mild perilobular fibrosis | 7 | 31 | 8 |
| Moderate and severe perilobular fibrosis | 23 | 85 | 20 |
|  |  |  |  |
| *Intralobular fibrosis* (*p* = 0.477) | | | |
| No and mild intralobular fibrosis | 8 | 45 | 11 |
| Moderate and severe intralobular fibrosis | 22 | 71 | 17 |
|  |  |  |  |
| *Acinar atrophy* (*p* = 0.516) | | | |
| No and mild atrophy | 8 | 44 | 11 |
| Moderate and severe atrophy | 22 | 73 | 17 |
|  |  |  |  |
| *Chronic inflammation* (*p* = 0.225) | | | |
| No and mild chronic inflammation | 19 | 89 | 18 |
| Moderate and severe chronic inflammation | 11 | 28 | 10 |
